# A Prospective Randomised Comparative Clinical Trial Study of Luteal Phase Letrozole versus Ganirelix Acetate Administration to Prevent Severity of Early Onset OHSS in ARTs

**DOI:** 10.22074/IJFS.2021.139562.1042

**Published:** 2021-10-16

**Authors:** Rana Afzal Choudhary, Priyanka H Vora, Kavita K Darade, Seema Pandey, Kedar N Ganla

**Affiliations:** 1 Ankoor Fertility Clinic, Mumbai, India; 2 Panache Hospital, Nashik, Maharashtra, India; 3 Seema Hospital and Eva Fertility Clinic, Atraulia, Uttar Pradesh, India

**Keywords:** Ganirelix Acetate, Gonadotrophin-Releasing Hormone Antagonist, *In Vitro* Fertilization, Letrozole, Ovarian Hyperstimulation Syndrome

## Abstract

**Background:**

Ovarian hyperstimulation syndrome (OHSS) is the most notable complication in ovulation induction for assisted reproductive techniques (ARTs) like *in vitro* fertilization (IVF) and intracytoplasmic sperm injection (ICSI). Hence, we decided to evaluate the effect of the aromatase inhibitor, letrozole, versus gonadotrophin-releasing hormone (GnRH)-antagonist (ganirelix acetate) on prevention of severity of OHSS and reduction in serum estradiol (E2) levels when administered during the luteal phase after oocyte retrieval in IVF/ICSI cycles.

**Materials and Methods:**

In this prospective single-centred, randomized, parallel-arm study, 122 patients were rand- omized to receive oral letrozole (n=61, 2.5 mg twice daily) or ganirelix acetate (n=61, 0.25 mg subcutaneously daily) from the day of egg retrieval for the next 7 days. Incidence and severity of early OHSS were the primary endpoints assessed by the signs, symptoms, and laboratory findings of OHSS (e.g., serum E2 levels). The secondary endpoints were patient satisfaction and the additional cost of therapy to prevent the severity of OHSS.

**Results:**

Letrozole group had lower incidence of OHSS (13.1%) compared to 19.6% in ganirelix acetate group (P=0.32). Serum E2 levels on post-pick up days 5 and 7 were significantly lower in the letrozole group when com- pared to the ganirelix acetate group (P=0.001). The majority of the patients in both groups had no major complica- tions. No significant difference was found between the study groups with respect to the incidence of OHSS (P=0.33). The additional cost per IVF cycle for prevention of severity of early-onset OHSS in the letrozole group was 5.32 USD compared to 267.26 USD in the ganirelix acetate group, which was almost fifty times costlier.

**Conclusion:**

Letrozole and ganirelix acetate have the same efficiency for the overall prevention of OHSS, whereas letrozole was more effective in preventing moderate OHSS. Letrozole had better patient satisfaction and is cheaper compared to GnRH antagonists (Registration number: CTRI/2020/10/028674).

## Introduction

In the latter part of the 20^th^ century, infertility treatment revolved around achieving controlled ovarian hyperstimulation along with *in vitro* fertilization (IVF) and intracytoplasmic sperm injection (ICSI) (1-4). Ovarian hyperstimulation syndrome (OHSS) is the most notable complication of ovulation induction in assisted reproductive technique (ART) (5). The contributing mechanism of OHSS is attributable to human chorionic gonadotropin (hCG) exposure either following ovulation trigger during IVF/ICSI (early OHSS) or by pregnancy achieved from embryo transfer (late OHSS) (6). The pathobiological basis for the prolonged luteotropic effect of hCG is a longer half-life of hCG (t1/2=24 to 36 hours) as compared to the luteinizing hormone (LH, t ½=20 minutes) (7). This is given to promote final follicular maturation prior to oocyte retrieval. OHSS is characterized by cystic enlargement of the ovaries and a fluid shift from the intravascular to the third space due to increased capillary permeability and ovarian neoangiogenesis.

The incidence of OHSS, as reported in the literature, varies from 3.1 to 6% for IVF cycles and increases proportionately depending on the risk profile of the patient (8, 9). Increased serum estradiol (E2) levels is an established risk factor for OHSS and hypercoagulability leading to further complications (10, 11). Depending on the clinical manifestation and laboratory findings, OHSS is classified as mild, moderate, and severe (12). Additionally, based on the timing of occurrence, OHSS is classified as early OHSS that occurs within 9 days of hCG trigger; and late OHSS is seen after 10 days of administering hCG (13).

Primary treatment strategies to prevent OHSS include appropriate modification of ovulation induction protocol after identifying appurtenant patient risk factors (14). Further, the use of gonadotrophin-releasing hormone (GnRH) antagonists after oocyte retrieval in the luteal phase has been promoted for reducing the severity of OHSS and has become increasingly popular (15). Recently, the use of letrozole, an oral aromatase inhibitor, during the luteal phase has been hypothesized as one of the strategies to prevent OHSS as it significantly reduces the E2 levels in the blood (16). Nonetheless, because of its shortened halflife and its effects on reproductive physiology, researchers are showing more interest in this drug. Moreover, randomized clinical trials have also claimed the efficacy of letrozole in reducing serum E2 levels (17). Existing studies on letrozole have confirmed its effectiveness by comparing it with either a placebo or an active comparator such as aspirin or Cetrorelix (16). In Wang et al. (16) study, a head-to-head comparison of letrozole to Cetrorelix (GnRH antagonist) showed no difference in the incidence of moderate to severe OHSS, hospitalization days, or duration of the luteal phase. Considering this, it is prudent to know the outcomes with letrozole and ganirelix acetate in the context of decreasing OHSS after ovulation trigger with an inducing agent, such as hCG or dual trigger during ART. Thus, we conducted a prospective comparative study to evaluate the effect of letrozole versus ganirelix acetate on serum E2 levels when administered during the luteal phase after oocyte retrieval in IVF/ICSI cycles for prevention and decreasing the severity of OHSS.

## Materials and Methods

### Study design

This prospective single-centred, randomized, parallel-arm study was conducted at a private reproductive medicine clinic for a period of one year from 16 October 2019. Ethics committee approval was obtained from Independent Ethics Committee after submitting the study related documents [Ethic committee Reg No: ECR/1.679/ Maruthi/lnd/ KA (2O18-Letter dated 16-Aug-2019)], and the study was registered in the Clinical Trials RegistryIndia [CTRI Registration No CTRI/2020/10/028674]. The study was conducted by adhering to all established norms of Good Clinical Practice (GCP) guidelines and Ethical principles laid down in the Indian Council for Medical Research guidelines for biomedical research on human participants, 2018.

### Study subjects

After obtaining written informed consent from 144 women, who are seeking ART, aged 20-30 years, with body mass index (BMI) between 18 to 29 kg/m^2^, basal levels of E2≤50 pg/mL on day 1 of stimulation, antiMüllerian hormone (AMH) >5 ng/ml, and antral follicle count (AFC) of >20 confirmed via ultrasound; and oocyte retrieval >25, serum E2 level >2500 pg/mL on the day of trigger and evidence of OHSS defined by documented clinical findings coupled with ultrasonographic evidence of ascites, or ovary diameter 10 cm on one or both sides, or puncture follicle number more than 30 (follicular diameter 14 mm on oocyte retrieval day) were recruited for the study. All women with serum E2 levels <2500 pg/mL on day of trigger and those who could not receive dual triggers (as outlined in the study subject section), coasting (withholding gonadotropin stimulation during controlled ovarian stimulation resulting in atresia of small follicles, or other preventive measures for managing OHSS), contraindications to letrozole or GnRH antagonist (ganirelix acetate), including severe liver and renal dysfunction were exempted.

### Study procedures

Screened couples were counseled regarding the risk and symptoms of OHSS before starting the enrolment. Participants could enter the study only once. Controlled ovarian stimulation was achieved using recombinant follicle-stimulating hormone (rFSH) 225 IU and GnRH antagonist 0.25 mg added from day 6 of their stimulation (fixed antagonist protocol). A dual trigger was given in the form of hCG 2000 IU and triptorelin acetate 0.2 mg. All embryos were cryopreserved on day 3 for transfer in future cycles (freeze all protocol).

### Intervention

Following administration of dual trigger, a total of 122 patients were randomized into letrozole and ganirelix acetate groups of 61 each. Letrozole group received 2.5 mg of oral letrozole (Letoval, Sun Pharma Laboratories Limited) twice daily. The ganirelix acetate group received ganirelix acetate 0.25 mg (Orgalutran, Merck Sharp, and Dohme) subcutaneously daily, from the day of egg retrieval for the next seven days. The computer randomization technique was used for randomization.

### Endpoints

The primary outcome was the incidence and severity of early OHSS measured by the symptoms, signs, and laboratory findings suggestive of OHSS, serum E2, and serum progesterone on the day of hCG/trigger administration, days 5 and 7 after ovum pick-up, and days for menses after oocyte retrieval. The secondary measurements were the patient satisfaction and additional cost of therapy for the prevention of OHSS. All the study participants received dopamine agonist (Cabergoline) 0.5 mg once daily from the day of trigger for the next 8 days as a conservative therapy for OHSS. The Short Assessment of Patient Satisfaction (SAPS) questionnaire was used to assess patient satisfaction (18).

### Criteria for the diagnosis and grading of ovarian hyperstimulation syndrome

- Mild OHSS: Abdominal bloating, mild abdominal pain, ovarian size usually < 8 cm
- Moderate OHSS: Moderate abdominal pain, nausea ± vomiting, ultrasound evidence of ascites, and ovarian size usually 8-12 cm
- Severe OHSS: Clinical ascites (± hydrothorax), oliguria (<300 ml/day or <30 ml/hour), haematocrit >0.45, hyponatraemia (sodium <135 mmol/L), hypo-osmolality (osmolality <282 mOsm/kg), hyperkalaemia (potassium >5 mmol/L), hypoproteinaemia (serum albumin <35 g/L), and ovarian size usually >12 cm
- Critical OHSS: Tense ascites/large hydrothorax, haematocrit > 0.55, white cell count > 25 000/ml, oliguria/anuria, thromboembolism, and acute respiratory distress syndrome

### Statistical analysis

A minimum sample of 60 patients was required for each arm to detect a significant difference by considering a two-sided t test at a significance level of 95% and power of 90%. Statistical analysis was performed using SPSS 21 (SPSS, Chicago, IL, USA). Categorical variables were represented in terms of percentages. The continuous variables with normal distribution were presented as mean ± standard deviation and compared using paired t test, whereas Chi-square test was employed for dichotomous data. Mann-Whitney U test was performed for variables without normal distribution. A P<0.05 was considered statistically significant at a 95% confidence interval.

## Results

**Fig 1:**
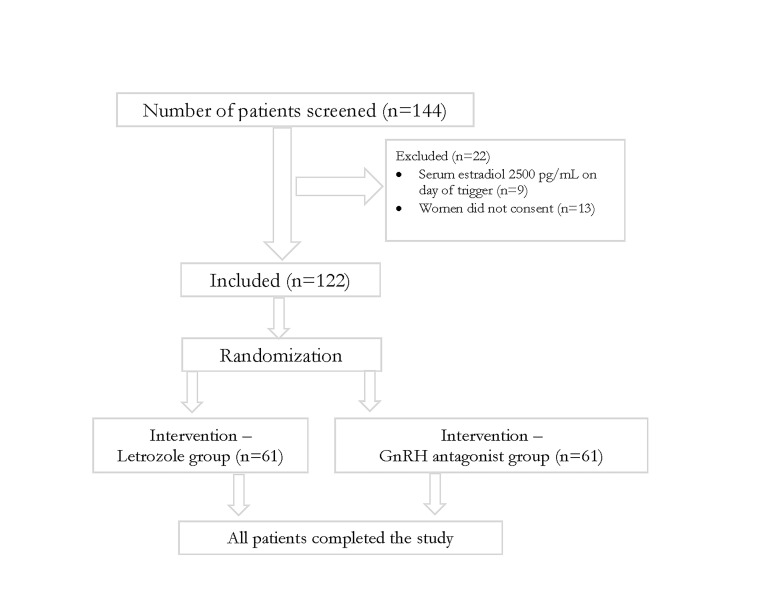
Consort diagram.

A total of 122 subjects were included in the study (letrozole group, n=61 and ganirelix acetate group, n=61). All the details are clearly depicted in the CONSORT diagram (Fig .1). Anovulation was the most commonly encountered cause for infertility, followed by polycystic ovary syndrome (PCOS) and male factor infertility. Baseline characteristics of the study population are presented in Table 1.

**Table 1:** Baseline characteristics of the study population


Parameters	Letrozole(n=61)	Ganirelix acetate(n=61)	P value^*^

Age (Y)	26.4 ± 2.4	26.8 ± 2.4	0.33^NS^
BMI (kg/m^2^)	22.3 ± 5.2	21.8 ± 5.9	0.65^NS^
Duration of infertility (Y)	4.3 ± 1.3	4.4 ± 1.2	0.78^NS^
Antral follicular count	26 ± 1.3	25 ± 1.8	> 0.05^NS^
Anti-mullerian hormone (ng/ml)	6.1 ± 1.3	5.9 ± 1.4	> 0.05^NS^
Follicle stimulating hormone (IU/L)	3.1 ± 1.1	3.2 ± 1.4	0.68^NS^
Luteinizing hormone (IU/L)	3.5 ± 1.3	3.4 ± 1.1	0.68^NS^
Oocytes retrieved	33.2 ± 15.4	34.9 ± 10	0.47^NS^
Metaphase II oocytes	25.7 ± 11.5	27.8 ± 8.5	0.23^NS^
Immature oocyte	3.2 ± 3.6	5.9 ± 10.7	0.06^NS^
Germinal vesicle	4.3 ± 3.7	5.6 ± 3.1	0.05^S^
Fertilised oocytes	24.9 ± 10.9	26.9 ± 8.4	0.26^NS^
Embryos cryopreserved (D3)	24.2 ± 10.9	19.2 ± 14.4	0.03^S^


Data are presented as mean ± standard deviation. *; Independent t test, BMI; Body mass index, NS; non-significant, and S; Significant.

**Fig 2:**
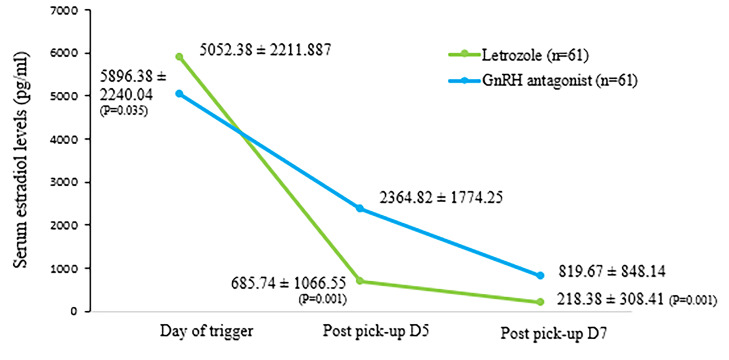
Mean serum estradiol levels between the study groups. D; Day and GnRH; Gonadotrophin-releasing hormone.

**Fig 3:**
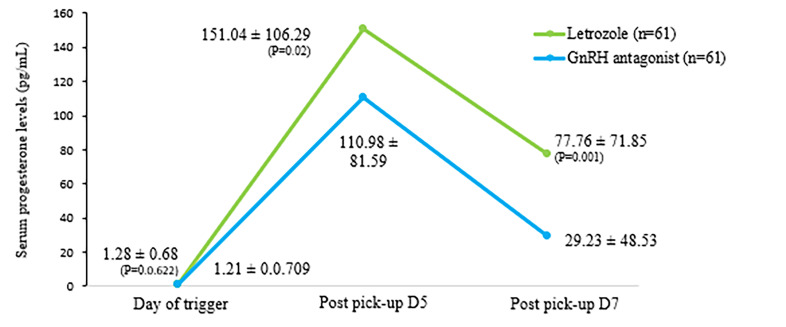
Mean serum progesterone levels between the study groups. D; Day and GnRH; Gonadotrophin-releasing hormone.

The letrozole group (6.2 ± 4.18 days) had early menses after oocyte retrieval as compared to the ganirelix acetate group (10.6 ± 1.3 days), which was statistically significant (P=0.001). On the trigger day, serum E2 levels were significantly different between letrozole and ganirelix acetate groups [844 pg/mL, P=0.04, 95% confidence interval (CI): 45.96 to 1642.04]. In addition, mean serum E2 levels were significantly lower in the letrozole group compared to the ganirelix acetate group on post-pick up days 5 and 7 (Fig .2). Figure 3 represents mean serum progesterone levels between the study groups on the day of trigger and post-pick up days 5 and 7.

**Fig 4:**
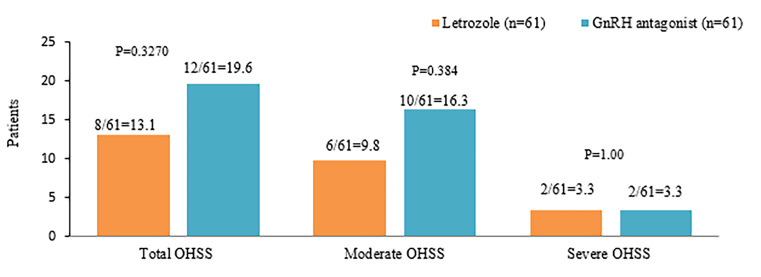
Distribution of OHSS between the study groups. OHSS; Ovarian hyperstimulation syndrome and GnRH; Gonadotrophin-releasing hormone.

**Fig 5:**
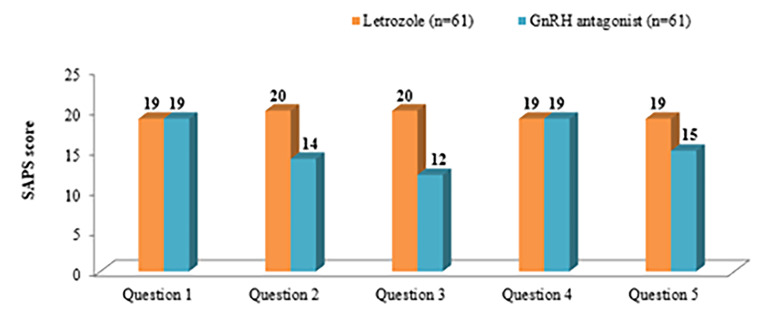
SAPS outcome score between the study groups. Q1; How satisfied are you with the explanations the doctor has given you about the your treatment?, Q2; Are you satisfied with the route of drugs (oral vs. injectable) being given to you?, Q3; Are you satisfied with the ease of taking these drugs (oral vs. injectable) being given to you?, Q4; Are you satisfied with the care you received in the clinic?, Q5; Are you satisfied with the overall treatment?, Scores: 1 to 5; Very dissatisfied, 5 to 10; Dissatisfied, 11 to 15; Satisfied, and 15 to 20; Very satisfied.

Statistically, no significant difference was found between the study groups with respect to the incidence of OHSS (P=0.33, Fig .4). According to the SAPS assessment, patients were 'very satisfied' with the route of administration and comfort of taking the treatment in the letrozole group compared to 'not so satisfied' in the ganirelix acetate group (20 vs. 14 and 20 vs. 12, respectively). A significant difference was observed between the study groups regarding patient satisfaction scores (Fig .5).

The additional cost per IVF/ICSI cycle for prevention of severity of early-onset OHSS in the letrozole group was 5.32 USD as compared to 267.26 USD in the ganirelix acetate group, which was almost fifty times costlier.

## Discussion

Currently, the management of patients undergoing IVF/ ICSI is centred on minimizing OHSS related complications while maintaining pregnancy efficacy outcomes. In the current study, mean serum E2 levels were significantly lower in the letrozole group compared to the ganirelix acetate group on post-pick up days 5 and 7. However, the majority of the patients in both groups had no significant complications.

In the current study, patients undergoing IVF/ICSI were treated during the luteal phase with either oral letrozole (aromatase inhibitors) or ganirelix acetate injection. In general, patients undergoing IVF have a drastic increase in their endogenous E2 levels starting with <60 pg/mL to >2500 pg/mL, or much higher if they develop OHSS. A high estrogenic milieu is a reasonable justification for forming a hypercoagulable state and puts patients at risk for venous or arterial thrombosis (18). Administration of letrozole during the luteal phase could be a new approach to reduce the risk of thrombosis associated with OHSS, as shown in the previous studies (19). Letrozole is an effective and extremely specific aromatase inhibitor. Letrozole hinders the aromatase enzyme by actively binding to the subunit cytochrome P450 of the enzyme resulting in an obstruction of androgen conversion into estrogen and reduces the risk of OHSS (20-22).

Published studies have proposed the use of letrozole would effectively reduce the E2 levels in patients with high risk for OHSS (16, 19). Similar findings were observed in the current study as well. As expected, letrozole has caused a more significant reduction in serum E2 levels in patients at high risk for OHSS in comparison to ganirelix acetate on day 7 post oocyte trigger, and a significant trend continued up to day 9. A similar trend was observed in Wang et al. (16) study carried out in 139 infertile women undergoing ART with high risk for OHSS. In that study, a significant decrease in the level of E2 was observed on the 4^th^, 7^th^, and 10^th^ days after hCG administration for letrozole (5 mg) compared to support therapy. In a similar study by Chen et al. (17) carried out in women with polycystic ovary syndrome (n=181), the letrozole-treated group had significantly lowered E2 levels on the day of retrieval as compared to the non-letrozole group (1001.60 vs. 1690.65 pg/ml). As compared to the study by Chen et al. (17), the BMI was the same in our study. However, there were differences in the AFC (~25 vs. 18) and AMH (~6 vs. 5 ng/mL, respectively) in our study. Ovarian response was also higher in our study as compared to the Chen et al. (17) study: the number of oocytes retrieved (33 vs. 18), the number of fertilized oocytes (24 vs. 13), and the number of embryos cryopreserved (19 vs. 6, respectively). The potential mechanism behind reducing the E2 levels with letrozole treatment could be attributed to either corpus luteum mediated hypothesis as a result of the luteolytic effect or vascular endothelial growth facto (VEGF) mediating downstream pathways or steroidogenic pathway as demonstrated by previous studies (16, 19, 20, 23, 24). This may limit the undesirable negative effect of accumulative E2 concentrations and prevent complications due to hypercoagulability and OHSS in these women (25-27).

Recently, letrozole was recommended as a preventive treatment in women at high risk for OHSS (28). Corresponding to this, in the current study, the incidence of OHSS was numerically less in the letrozole group than the ganirelix group, although statistically non-significant (13.1 vs. 19.6%). Besides, letrozole was better than GnRH antagonist in preventing moderate OHSS (9.8 vs. 16.3%), though non-significant. Although Chen et al. (17), in their study demonstrated a decrease in the incidence rate of OHSS in the letrozole-treated group compared to the nonletrozole group (2.56 vs. 7.77%), the differences were not significant, possibly because of the small study sample size. Our findings comply with Mai et al.'s (29) findings, who compared the effectiveness of letrozole with aspirin in preventing early OHSS (in the luteal phase). This study has demonstrated less incidence of OHSS in patients receiving letrozole compared to aspirin, which could be credited to luteolysis rather than the VEFG effects.

On the other hand, with progesterone, we observed an initial rise to 5 days, after which the levels witnessed a sharp decline at the end of day 7. Similar patterns of a peak at day 5 followed by a fall by day 7 were observed in the two studies reported by Lainas et al. (15, 30). This distinct pattern of a rise in progesterone is because of the luteotropic effect of hCG, which mimics LH but with a longer half-life of HCG up to 36 hours (7). Given the hormonal findings, it is reasonable to conclude that the aromatase enzyme inhibition by letrozole causes a significantly greater decrease in serum E2 compared to blocking the hypothalamic-pituitary-gonadal pathway by ganirelix. The smaller but significantly higher levels of progesterone require further validation.

In this study, no complications were observed in most participants; however, moderate OHSS was observed in a limited number of patients in the letrozole group compared to the ganirelix group. Additionally, participants in the letrozole group had early menses after oocyte retrieval compared to the ganirelix acetate group, and the difference noticed was -4.41 days.

Considering the results of the study, it is imperative to discuss the pathophysiological mechanisms for the beneficial effects observed in our study. Two main hypotheses are proposed for the development of OHSS: first, estrogen-mediated- as studies have shown that patients with high E2 levels of >2500 pg/mL are at increased risk of OHSS (10); however, there have been conflicting reports regarding the estrogen hypothesis. Second, corpus luteum mediated hypothesis due to the luteotropic effect, with VEGF mediating downstream pathways (16, 19). Letrozole increases local androgen levels and thereby influences the granulosa lutein cells to decrease VEGF and E2 levels (19, 28). Patients receiving letrozole have a shorter luteal phase and lower VEGF levels, indicating the corpus luteum pathway is a more plausible hypothesis. However, as research in this field is at a nascent stage, further studies are required in this regard.

Lastly, our study showed that patients prefer oral therapy due to ease of administration and the lower cost associated with the intervention. The cost of treatment with letrozole is significantly less than ganirelix (5.32 USD vs. 267.26 USD). Therefore, in a resource-constrained setting and a developing country like India, more patients would be able to afford the intervention and benefit from this therapy, making this approach patient-friendly in selected cases.

Although to the best of our knowledge, we are the first to report a head-to-head comparison between letrozole and ganirelix in mitigating OHSS symptoms when these agents are administered post-oocyte retrieval, our study has few limitations. Firstly, this was a singlecentred unblinded trial design; hence it will not give a comprehensive view of the entire population. Secondly, we did not stratify patients with higher baseline E2 levels. This is a known risk factor for developing OHSS and could have confounded our results. Thirdly, other outcomes such as VEGF levels would have been worth exploring to understand the outcomes truly.

## Conclusion

Letrozole and ganirelix acetate have the same efficiency for the overall prevention of OHSS, among which letrozole was more effective in preventing moderate OHSS. Letrozole had better patient satisfaction and was cheaper compared to GnRH antagonists. In the future, rigorous randomized trials are required to evaluate the effect of letrozole and its endocrine impact on the development of OHSS.

## References

[1] Beall SA, DeCherney A (2012). History and challenges surrounding ovarian stimulation in the treatment of infertility. Fertil Steril.

[2] Depalo R, Jayakrishan K, Garruti G, Totaro I, Panzarino M, Giorgino F (2012). GnRH agonist versus GnRH antagonist in in vitro fertilization and embryo transfer (IVF/ET).. Reprod Biol Endocrinol.

[3] Erb K, Klipping C, Duijkers I, Pechstein B, Schueler A, Hermann R (2001). Pharmacodynamic effects and plasma pharmacokinetics of single doses of cetrorelix acetate in healthy premenopausal women. Fertil Steril.

[4] Song M, Liu C, Hu R, Wang F, Huo Z (2020). Administration effects of single-dose GnRH agonist for luteal support in females undertaking IVF/ICSI cycles: a meta-analysis of randomized controlled trials. Exp Ther Med.

[5] Blumenfeld Z (2018). The ovarian hyperstimulation syndrome. Vitam Horm.

[6] Nastri CO, Teixeira DM, Moroni RM, Leitão VM, Martins WP (2015). Ovarian hyperstimulation syndrome: pathophysiology, staging, prediction and prevention. Ultrasound Obstet Gynecol.

[7] Choi J, Smitz J (2014). Luteinizing hormone and human chorionic gonadotropin: distinguishing unique physiologic roles. Gynecol Endocrinol.

[8] Peigne M, Lobert M, Tintillier V, Trillot N, Catteau-Jonard S, Dewailly D (2017). Prevalence of ovarian hyperstimulation syndrome (OHSS) and hypercoagulability in patients triggered by GnRH agonist for excessive follicular response: a systematic follow-up. Fertil Steril.

[9] Toftager M, Bogstad J, Bryndorf T, Løssl K, Roskær J, Holland T (2016). Risk of severe ovarian hyperstimulation syndrome in GnRH antagonist versus GnRH agonist protocol: RCT including 1050 first IVF/ICSI cycles. Hum Reprod.

[10] D'Angelo A, Davies R, Salah E, Nix BA, Amso NN (2004). Value of the serum estradiol level for preventing ovarian hyperstimulation syndrome: a retrospective case control study. Fertil Steril.

[11] Corbett S, Shmorgun D, Claman P (2014). Reproductive Endocrinology Infertility Committee; Special Contributor.The prevention of ovarian hyperstimulation syndrome. J Obstet Gynaecol Can.

[12] Namavar Jahromi B, Parsanezhad ME, Shomali Z, Bakhshai P, Alborzi M, Moin Vaziri N (2018). Ovarian hyperstimulation syndrome: a narrative review of its pathophysiology, risk factors, prevention, classification, and management. Iran J Med Sci.

[13] Huang H, Takai Y, Samejima K, Narita T, Ichinose S, Itaya Y (2020). Late-onset ovarian hyperstimulation syndrome developing during ovarian stimulation in an ectopic pregnancy: a case report. J Med Case Rep.

[14] Bosch E, Ezcurra D (2011). Individualised controlled ovarian stimulation (iCOS): maximising success rates for assisted reproductive technology patients. Reprod Biol Endocrinol.

[15] Lainas GT, Kolibianakis EM, Sfontouris IA, Zorzovilis IZ, Petsas GK, Tarlatzi TB (2012). Outpatient management of severe early OHSS by administration of GnRH antagonist in the luteal phase: an observational cohort study. Reprod Biol Endocrinol.

[16] Wang Yq, Yang J, Xu Wm, Xie Qz, Yan Wj, Yin Tl, et al (2013). Luteal letrozole administration decreases serum estrogen level but not the risk of ovarian hyperstimulation syndrome. Beijing Da Xue Xue Bao Yi Xue Ban.

[17] Chen Y, Yang T, Hao C, Zhao J (2018). A retrospective study of letrozole treatment prior to human chorionic gonadotropin in women with polycystic ovary syndrome undergoing in vitro fertilization at risk of ovarian hyperstimulation syndrome. Med Sci Monit.

[18] Hawthorne G, Sansoni J, Hayes L, Marosszeky N, Sansoni E (2014). Measuring patient satisfaction with health care treatment using the short assessment of patient satisfaction measure delivered superior and robust satisfaction estimates. J Clin Epidemiol.

[19] Garcia-Velasco JA, Quea G, Piró M, Mayoral M, Ruiz M, Toribio M (2009). Letrozole administration during the luteal phase after ovarian stimulation impacts corpus luteum function: a randomized, placebo-controlled trial. Fertil Steril.

[20] Garcia-Velasco JA, Moreno L, Pacheco A, Guillén A, Duque L, Requena A (2005). The aromatase inhibitor letrozole increases the concentration of intraovarian androgens and improves in vitro fertilization outcome in low responder patients: a pilot study. Fertil Steril.

[21] Pereira N, Hancock K, Cordeiro CN, Lekovich JP, Schattman GL, Rosenwaks Z (2016). Comparison of ovarian stimulation response in patients with breast cancer undergoing ovarian stimulation with letrozole and gonadotropins to patients undergoing ovarian stimulation with gonadotropins alone for elective cryopreservation of oocytes. Gynecol Endocrinol.

[22] Goldrat O, Gervy C, Englert Y, Delbaere A, Demeestere I (2015). Progesterone levels in letrozole associated controlled ovarian stimulation for fertility preservation in breast cancer patients. Hum Reprod.

[23] Danastas K, Whittington CM, Dowland SN, Combes V, Murphy CR, Lindsay LA (2019). Ovarian hyperstimulation reduces vascular endothelial growth factor-A during uterine receptivity. Reprod Sci.

[24] He Q, Liang L, Zhang C, Li H, Ge Z, Wang L (2014). Effects of different doses of letrozole on the incidence of early-onset ovarian hyperstimulation syndrome after oocyte retrieval. Syst Biol Reprod Med.

[25] Azmoodeh A, Pejman Manesh M, Akbari Asbagh F, Ghaseminejad A, Hamzehgardeshi Z (2015). Effects of letrozole-HMG and clomipheneHMG on incidence of luteinized unruptured follicle syndrome in infertile women undergoing induction ovulation and intrauterine insemination: a randomised trial. Glob J Health Sci.

[26] D'Amato G, Caringella AM, Stanziano A, Cantatore C, Palini S, Caroppo E (2018). Mild ovarian stimulation with letrozole plus fixed dose human menopausal gonadotropin prior to IVF/ICSI for infertile nonobese women with polycystic ovarian syndrome being pre-treated with metformin: a pilot study. Reprod Biol Endocrinol.

[27] Behnoud N, Farzaneh F, Ershadi S (2019). The effect of clomiphene citrate versus letrozole on pregnancy rate in women with polycystic ovary syndrome: a randomized clinical trial. Crescent J Medical Biol Sci.

[28] Wang YQ, Luo J, Xu WM, Xie QZ, Yan WJ, Wu GX (2015). Can steroidal ovarian suppression during the luteal phase after oocyte retrieval reduce the risk of severe OHSS?. J Ovarian Res.

[29] Mai Q, Hu X, Yang G, Luo Y, Huang K, Yuan Y (2017). Effect of letrozole on moderate and severe early-onset ovarian hyperstimulation syndrome in high-risk women: a prospective randomized trial. Am J Obstet Gynecol.

[30] Lainas GT, Kolibianakis EM, Sfontouris IA, Zorzovilis IZ, Petsas GK, Lainas TG (2013). Pregnancy and neonatal outcomes following luteal GnRH antagonist administration in patients with severe early OHSS. Hum Reprod.

